# Emerging functions of pannexin 1 in the eye

**DOI:** 10.3389/fncel.2014.00263

**Published:** 2014-09-15

**Authors:** Sarah Kurtenbach, Stefan Kurtenbach, Georg Zoidl

**Affiliations:** ^1^Department of Psychology, Faculty of Health, York UniversityToronto, ON, Canada; ^2^Department of Biology, Faculty of Science, York UniversityToronto, ON, Canada

**Keywords:** pannexin 1 (Panx1), eye, retina, lens, cornea, purinergic receptor signaling, ATP, feedback mechanism

## Abstract

Pannexin 1 (Panx1) is a high-conductance, voltage-gated channel protein found in vertebrates. Panx1 is widely expressed in many organs and tissues, including sensory systems. In the eye, Panx1 is expressed in major divisions including the retina, lens and cornea. Panx1 is found in different neuronal and non-neuronal cell types. The channel is mechanosensitive and responds to changes in extracellular ATP, intracellular calcium, pH, or ROS/nitric oxide. Since Panx1 channels operate at the crossroad of major signaling pathways, physiological functions in important autocrine and paracrine feedback signaling mechanisms were hypothesized. This review starts with describing in depth the initial Panx1 expression and localization studies fostering functional studies that uncovered distinct roles in processing visual information in subsets of neurons in the rodent and fish retina. Panx1 is expressed along the entire anatomical axis from optical nerve to retina and cornea in glia, epithelial and endothelial cells as well as in neurons. The expression and diverse localizations throughout the eye points towards versatile functions of Panx1 in neuronal and non-neuronal cells, implicating Panx1 in the crosstalk between immune and neural cells, pressure related pathological conditions like glaucoma, wound repair or neuronal cell death caused by ischemia. Summarizing the literature on Panx1 in the eye highlights the diversity of emerging Panx1 channel functions in health and disease.

## Introduction

In the year 2000, pannexin (Panx) genes (Panx, Latin: *pan* = complete, everywhere and *nexus* = junction) were described as putative gap junction proteins based on a distant sequence homology to innexins, the gap junction proteins of invertebrates. In higher vertebrates, the Panx gene family consists of the three glycosylated integral membrane proteins Panx1, Panx2 and Panx3 (Panchin et al., 2000; Bruzzone et al., 2003; Baranova et al., 2004). Although, initial studies suggested that under certain conditions pannexins might be able to form gap junction channels *in vitro* (Bruzzone et al., 2003, 2005; Vanden Abeele et al., 2006; Lai et al., 2007), experimental evidence suggests that pannexins function as unopposed single membrane channels *in vivo* (Sosinsky et al., 2011).

Today, Panx1 is the best-characterized family member. Panx1 is almost ubiquitously expressed and found in many organs as well as in several cell types of the blood and immune system (Dvoriantchikova et al., 2006a,b; Locovei et al., 2006a; Penuela et al., 2007; Schenk et al., 2008; Seminario-Vidal et al., 2009, 2011; Celetti et al., 2010; Sridharan et al., 2010; Woehrle et al., 2010; Kienitz et al., 2011; Hanner et al., 2012). In the CNS, Panx1 shows a widespread distribution and largely co-localizes with Panx2 (Bruzzone et al., 2003; Ray et al., 2005; Vogt et al., 2005; Dvoriantchikova et al., 2006a,b; Ray et al., 2006; Zappalà et al., 2006, 2007), where the expression is mainly neuronal (Ray et al., 2005, 2006; Zoidl et al., 2007). Evidence for glial expression was found in cultured astrocytes and oligodendrocytes (Boassa et al., 2007; Huang et al., 2007a; Iglesias et al., 2009a; Suadicani et al., 2012). Further, Panx1 expression has been described in major sensory systems including the eye, inner ear, taste buds, and the olfactory epithelium (Bruzzone et al., 2003; Huang et al., 2007b; Romanov et al., 2007; Tang et al., 2008; Dando and Roper, 2009; Wang et al., 2009; Zhang et al., 2012). Panx2 is expressed in the eye, thyroid, kidney and liver, with highest expression levels in the brain and spinal cord (Bruzzone et al., 2003; Baranova et al., 2004; Vogt et al., 2005; Dvoriantchikova et al., 2006a,b; Ray et al., 2006). In humans, Panx2 expression is presumably brain specific. Panx3 is mainly expressed in the skin and cartilage, but can also be found in the heart ventricle, cochlea as well as in lung, kidney, thymus, liver and spleen and possibly astrocytes (Bruzzone et al., 2003; Penuela et al., 2007; Wang et al., 2009; Celetti et al., 2010).

Panx1 forms large-conductance channels, activated by changes in membrane potential, ATP, intracellular calcium, stretch, pH, elevated extracellular potassium, and following purinergic receptor activation (Bao et al., 2004a; Locovei et al., 2006a,b; Qiu and Dahl, 2009; Kawamura et al., 2010; Kienitz et al., 2011; Qiu et al., 2011; Kurtenbach et al., 2013). Channel opening and closing are subject to a plethora of molecular mechanisms including protein interactions, and post-translational modifications like glycosylation and S-nitrosylation (Johnstone et al., 2012; Lohman et al., 2012b; D’hondt et al., 2013; Penuela et al., 2014b; Retamal, 2014). Since Panx1 channels operate at the crossroad of major signaling pathways, foremost those involving intracellular calcium, extracellular ATP or ROS/nitric oxide, physiological functions in important autocrine and paracrine feedback signaling mechanisms were hypothesized (Kawamura et al., 2010; Kronlage et al., 2010; Lohman et al., 2012a; Bao et al., 2013). Further interest in Panx1 derives from independent lines of evidence supporting a critical role of Panx1 in pathological conditions. There, opening of Panx1 channels, frequently together with activation of purinergic receptors by extracellular ATP, has been implicated in diverse conditions including, but not limited to, ischemia (Madry et al., 2010; Monaco and Friedlander, 2012), trauma (Minkiewicz et al., 2013), seizures (Zappalà et al., 2006; Thompson et al., 2008; Mylvaganam et al., 2010; Kim and Kang, 2011; Santiago et al., 2011), HIV infection (Séror et al., 2011; Orellana et al., 2013), inflammation (Kanneganti et al., 2007; Schenk et al., 2008; de Rivero Vaccari et al., 2009; Lamkanfi et al., 2009; Schalper et al., 2009; Silverman et al., 2009), migraine (Karatas et al., 2013), and tumor cell growth (Lai et al., 2007; Penuela et al., 2012).

Important developments in Panx research and emerging roles in health and disease have been covered recently in excellent reviews (MacVicar and Thompson, 2010; Dahl and Keane, 2012; Bond and Naus, 2014; Penuela et al., 2014b). This review will address emerging roles of Panx1 in the eye. The anatomical axis of the eye is used to guide the reader through each chapter. Original studies demonstrating the expression and localization of Panx1 in neuronal and non-neuronal cell types throughout the eye are summarized (overview see Table 1). Furthermore, known functions are described, otherwise emerging roles of Panx1 in physiological and pathological conditions are highlighted and challenges outlined. In concluding remarks, functional diversity is discussed as the overarching theme. Readers interested in the roles of connexins in the eye can consult recently published reviews (Volgyi et al., 2013; Vroman et al., 2013; Berthoud et al., 2014; Beyer and Berthoud, 2014).

**Table 1:** **Summary of known and emerging functions of pannexins in the eye**.

Organism	Protein	Localization	Cell type	Localization	Interaction/Pathway	Function
mouse, rat	Panx1	retina: GCL, IPL, INL, ONL, OPL	RGC	neuronal processes	n.d.	neuroprotection, ischemia induced cell death (Dvoriantchikova et al., 2012)
					P2X7-R, purinergic signaling pathway	activation of P2X7-R by hypotonicity-incuded ATP release, glaucoma *
			HC	dendrites, invaginating rod spherules	n.d.	n.d.
			CB3a bipolar cells	soma, dendritic branches, axons	P2X7-R, purinergic signaling pathway	ATP release, photoreceptor-HC feedback regulation, neuronal processing (Kranz et al., 2013)
			amacrine cells	n.d.	n.d.	n.d.
			microglia	n.d.	AMPA/kainate receptor	ATP release
		lens	epithelial cells	predominantly in cytoplasm	n.d.	n.d.
			young elongated fiber cells	predominantly in cytoplasm	n.d.	n.d.
			mature fiber cells	plasma membrane	n.d.	n.d.
		tunica vasculosa lentis	endothelial cells	n.d.	n.d.	retinopathy of prematurity *
		cornea	epithelial cells		P2X7-R, purinergic signaling pathway	wound repair *
	Panx2	retina: all layers	n.d.	n.d.	n.d.	n.d.
		lens	epithelium and fiber cells	n.d.	n.d.	n.d.
zebrafish	drPanx1a	retina: OPL	HC	mainly cone synaptic complex, soma	n.d.	pH dependent photoreceptor-HC feedback mechanism, ATP release, neuronal processing (Vroman et al., 2014)
	drPanx1b	retina: GCL, INL	amacrine cells*	n.d.	n.d.	n.d.
	drPanx2	Retina	n.d.	n.d.	n.d.	n.d.
porcine	Panx1	lens	cuboidal epithelial cells	n.d.	Na^+^/K^+^-ATPase, P2Y-R, TRPV4	pressure regulation 1. Hyposmotic stress mediated ATP release 2. Hyposmotic stress mediated Ca^2+^ influx activates Panx1 mediating
bovine	Panx1	trabecular meshwork cells	n.d.	n.d.	P2X7-R, connexins	pressure-activated ATP release, glaucoma*
		ciliary epithelial cells	n.d	n.d.	connexins, P2X7-R and independent vesicular ATP release	hypotonicity-triggered ATP release

*n.d. indicates category not determined*.

* indicates emerging functions or indirect association with disease/disorders.

## Panx1 in the rodent retina: processing visual information

After the initial discovery of Panx expression in the eye (Bruzzone et al., 2003), refined characterizations of the rodent retina using *in situ* *hybridization* analysis and RT-PCR analysis of tissue captured by laser microdissection revealed Panx1 and Panx2 expression in the ganglion cell layer (GCL), inner nuclear layer (INL), the outer nuclear layer (ONL) and to a lesser extent in the inner plexiform layer (IPL; Ray et al., 2005; Dvoriantchikova et al., 2006a). In general, two Panx1 protein isoforms (43 kDa, 58 kDa) were found in the retina and brain, of which the 58 kDa variant is post-translationally modified (Kranz et al., 2013). RT-PCR and immunohistochemistry (IHC) revealed mRNA and protein expression regulation during development with a transient expression peak around birth, declining with age (Ray et al., 2005; Dvoriantchikova et al., 2006a). In neonatal and P20 animals, Panx1 labeling was prominent in retinal ganglion cells (RGC), amacrine cells and horizontal cells (HC) in the outer plexiform layer (OPL), whereas in adult animals the labeling mainly occurred in RGCs. Panx1 proteins were localized at the cell surface and neuronal processes.

More detailed analyses of mice retinae revealed Panx1 proteins in a puncta-like pattern on the soma and dendritic branches, sparsely along the axon, and more intensively at the axon terminal of type 3a OFF bipolar cells (CB3a cells) and on non-invaginating cellular processes at the base of rod spherules, resembling the flat contact between rods and cone bipolar cells. The authors also confirmed the expression of Panx1 in HC using single cell RT-PCR, IHC and ultrastructural analysis and found Panx1 localized at the tips of HC dendrites invaginating in rod spherules. It is worth noting that only one HC type is present in the murine retina receiving cone input at the dendrites and rod input at the axon terminals (Peichl and Gonzalez-Soriano, 1993) in contrast to the zebrafish retina possessing two types of HC that either make contacts to rods or cones, with the zebrafish drPanx1a (see below) found only at the HC cone synaptic complexes (Prochnow et al., 2009). In mice, the P2X7 receptor (P2X7-R), like Panx1, is localized at the tip of HC dendrites (Puthussery et al., 2006), suggesting that P2X7-R and Panx1 functions could be linked and involve the local release of ATP.

Physiological function(s) of Panx1 in the processing of visual information were investigated using Panx1^−/−^ mice, using *in vivo* and *in vitro* electroretinography (ERG). Kranz et al. (2013) demonstrated that under scotopic, but not photopic light conditions, the a- and b-wave amplitude of *in vivo* ERGs was increased in knockout animals at high light intensity. *In vitro* studies confirmed this result, and it was concluded that the ablation of Panx1 interferes with the activity of the dark-adapted retina without altering the temporal properties of signal transmission within the retina, but does not affect the cone pathway under light conditions. The authors proposed that Panx1 closes under conditions of prolonged light adaptation as previously observed for retinal gap junctions (Bloomfield et al., 1995; Xin and Bloomfield, 1999). In scotopic light conditions, the changes to the b-wave were explained by the Panx1 channels on the CB3a cells providing a competing current to the light-evoked current of the ON bipolar cells. This might lead to a Panx1-mediated reduction of radial current flow, resulting in an enhanced b-wave in the Panx1^−/−^ mice. The a-wave reflects the reduction of the dark current during light stimulation and is made up of an extracellular radial current from the photoreceptor inner segment towards the outer segments (Penn and Hagins, 1969) and from currents arising from the photoreceptor synapse (Hagins et al., 1970). In this context, Panx1 channels on HC dendrites may take part in negative feedback mechanisms leading to a decreased current flow at the photoreceptor synapse, causing an enlarged a-wave if Panx1 channels are not present. A mechanism addressing this feedback has been previously put forward for connexin hemichannels in the fish and turtle retina and new insight will follow below (Kamermans et al., 2001; Pottek et al., 2003; Kamermans and Fahrenfort, 2004).

## Panx1 orthologs in the fish retina: feedback regulation in the outer retina

The retina of zebrafish shares many properties with those of higher vertebrates, but color vision is more similar to the human trichromatic vision when compared to the murine dichromatic vision (Goldsmith and Harris, 2003; Conway, 2007). Series of genome duplication events in teleost evolution caused partial gene duplications approximately 320–350 million years ago (Jaillon et al., 2004; Postlethwait, 2007; Ravi and Venkatesh, 2008). As a consequence, the zebrafish genome encodes for two Panx1 genes, drPanx1a and drPanx1b (Bond et al., 2012; Kurtenbach et al., 2013), and single drPanx2 and drPanx3 genes. In the zebrafish retina, drPanx1a, drPanx1b and three differently spliced mRNA transcripts of drPanx2 are expressed (Zoidl et al., 2008; Prochnow et al., 2009; Kurtenbach et al., 2013). The retinal expression of drPanx1a and drPanx1b has been further characterized using IHC. drPanx1a was found in a band-like, horseshoe shape pattern in the OPL on HC dendrites, but also on HC somata (Prochnow et al., 2009; Kurtenbach et al., 2013). No labeling could be found on other cell types like ON bipolar cells or interplexiform cells. Ultrastructural analysis revealed the expression of drPanx1a on HC dendrites inserted deeply in the cone synaptic complex, where they are located more distal from the cones’ glutamate release sites compared to Cx55.5 hemichannels. No expression in rod synaptic terminals was found. IHC located drPanx1b in the GCL and in the INL suggestive for a potential localization in amacrine cells (Kurtenbach et al., 2013). Like mammalian Panx1, drPanx1a and drPanx1b are glycoproteins, with drPanx1b having three possible N-glycosylation sites and drPanx1a one. Fish pannexins, like mammalian Panx channels, form voltage-gated single membrane channels with shared and unique properties, including activation under physiological conditions, modulation by extracellular ATP, intracellular Ca^2+^ and pH changes (Kurtenbach et al., 2013).

Exciting novel results highlighted the role of drPanx1a in neuronal processing at the first retinal synapse, where HCs inhibit photoreceptors. This interaction generates the center/surround organization of bipolar cell receptive fields and is crucial for contrast enhancement (Burkhardt and Fahey, 1998; Jackman et al., 2011). Two competing hypotheses considered an ephaptic or a proton-mediated mechanism to explain this fundamental process (Kamermans et al., 2001; Kamermans and Fahrenfort, 2004; Fahrenfort et al., 2009; Hirasawa et al., 2012; Klaassen et al., 2012; Vroman et al., 2013). Since zebrafish with a functional knock out of the HC specific Cx55.5 retained 40% hemichannel conductance, it was tempting to speculate that other connexins or a Panx could account for the residual 40% conductance (Klaassen et al., 2011; Sun et al., 2012). The Kamermans group was able to answer this long-standing, fundamental question demonstrating that HC feedback to photoreceptors via an unexpected synthesis of both mechanisms (Vroman et al., 2014). The first one is a very fast connexin driven ephaptic mechanism, which has no synaptic delay making it one of the fastest inhibitory synapses known. The second one is a relatively slow mechanism, depending on ATP released via Panx1 channels located on horizontal cell dendrites deeply invaginating the cone synaptic terminal.

The unique finding was that the extracellular ATP hydrolysis to AMP, phosphate groups and protons, formed a buffer with a pKa of 7.2. This inhibited Ca^2+^-channels in cones, and consequently reduced the cones’ glutamate release. Since cone photoreceptors and HCs form a reciprocal synapse with cones transmitting to HCs through an excitatory synapse and HCs feeding back to cones through an inhibitory synapse, the reduction of glutamate release caused hyperpolarization in HCs, a condition that evoked a decrease of Panx1 channels conductance. Decreasing ATP release caused alkalization in the synaptic cleft and consequently increased cone glutamate release. Surprisingly, the hydrolysis of ATP instead of ATP itself mediated the synaptic modulation, revealing a novel form of synaptic modulation. Since Panx1 channels and ecto-ATPases are strongly expressed in the nervous system and Panx1 function was previously implicated in synaptic plasticity, learning and behavior (Prochnow et al., 2012), the authors hypothesized that this novel form of synaptic modulation might be a wide spread phenomenon.

## Panx1 in retinal microglia: implications for crosstalk of immune and neural cells

Panx1-mediated ATP release has been associated with the regulation of the morphology and behavior of “resting” microglia in the retina, the primary resident immune cells in the CNS, because of endogenous, ionotropic glutamatergic neurotransmission (Fontainhas et al., 2011). In their “resting” state, microglia have a ramified morphology with fine, extended processes, which exhibit rapid dynamics to make repeated contacts with neurons, glia and blood vessels (Davalos et al., 2005; Nimmerjahn et al., 2005; Lee et al., 2008). The factors that regulate “resting” microglia and induce their transformation to the “activated” state are not fully understood, but a credible hypothesis is that gradients of “on” and “off” signals activate/repress microglial activation involving extracellular signals like ATP, purinergic receptors, glutamate receptors, chemokines and neurotransmission (Mertsch et al., 2001; Xiang et al., 2006; Liang et al., 2010; Wong et al., 2011; Domercq et al., 2013). Experimental evidence suggesting Panx1 involvement derived from *ex vivo* retinal mouse explants, pharmacological intervention of retinal neurotransmission and analysis of microglia morphology (Fontainhas et al., 2011). Microglial morphology and dynamic behavior was modulated by retinal neurotransmission. Endogenous ionotropic glutamate through AMPA/kainate receptors maintained and increased dendritic morphology and process motility, whereas ionotropic GABAergic neurotransmission negatively regulated the morphology and motility. Since the application of probenecid decreased the AMPA mediated effects on microglia, the authors concluded that the response to neurotransmitter was mediated indirectly via secondary ATP, released in response to glutamatergic neurotransmission through probenecid-sensitive Panx channels. Interestingly, the effects of fast AMPA/kainate receptors on microglia were larger compared to those of slow NMDA receptors, although NMDA receptor stimulation has been shown triggering Panx1 opening in pyramidal neurons leading to epileptiform seizure activity (Thompson et al., 2008). This mode of constitutive signaling between neural and immune cells of the CNS illustrates the versatility of Panx1 channels for distinct physiological processes in the retina and beyond.

## Panx1 in retinal ganglion cells: implications for ischemic disorders of the retina

A role of Panx1 channels in the pathology of the retina was investigated using conditional Panx1^−/−^ mice and oxygen-glucose deprivation (OGD) to demonstrate whether Panx1 is part of a pathological cascade leading to ischemia-induced RGC loss (Dvoriantchikova et al., 2012). They found that Panx1 deficiency protects RGCs from death induced by ischemia, most likely by preventing rapid membrane permeation and Panx1 activation as part of the activation of neuronal inflammasome. It was concluded that Panx1 is activated at a convergence point for several neurotoxic pathways. Further, it was suggested that the Panx1 channel is a potential target for therapeutic intervention in retinal ischemic disorders.

## Panx1 in the lens: implications for a role in hypoosmotic stress

The vertebrate lens is an avascular tissue with extensive gap junctional and hemichannel coupling supporting growth, differentiation and homeostasis. Using qRT-PCR and *in situ* hybridization, Dvoriantchikova et al. (2006b) demonstrated Panx1 and Panx2 mRNA expression in the lens epithelium and fiber cells. Relative to Cx50, the most abundant connexin expressed in the mouse lens, Panx1 and Panx2 expression is significantly lower in the epithelium and the fiber cells. Western blot analyses revealed four Panx1 protein isoforms: two major protein bands (43 and 120 kDa) and two minor bands (58 and 62 kDa). Using differential centrifugation, the monomeric 43 kDa protein was found in the soluble protein fraction, whereas the 62 kDa variant was present in the microsomal, organelle-enriched fraction, likely associated with the ER and Golgi apparatus. Further detergent extraction with Triton X-100 and methyl-β-cyclodextrin suggested association of the 58 kDa and 120 kDa isoforms with lipid raft membrane microdomains. The water-insoluble Panx1 62 and 120 kDa isoforms were lens and retina specific, whereas 58 kDa species is found in non-CNS tissues. Expression was age and cell type dependent, with the 120 kDa protein predominantly expressed in mature and young fibers, whereas the 58 kDa species was mainly present in the lens epithelium and the young elongating fibers. IHC analysis revealed that Panx1 proteins were predominantly located in the lens cortex and that expression declined towards the lens nucleus. The subcellular distribution was consistent with the expected live cycle of the Panx1 channel protein. During development, redistribution occurred shifting from a predominant presence in the cytoplasm in epithelial and young elongated fiber cells to a more pronounced localization in the plasma membrane of mature fiber cells.

Potential function(s) of Panx1 in the lens epithelium were investigated using the porcine lens model (Shahidullah et al., 2012). In the lens the cuboidal lens epithelium is located in the anterior portion between the lens capsule and the lens fibers regulating important homeostatic functions and contains a variety of transporters as well as, amongst others, purinergic receptors (Delamere and Tamiya, 2009). Panx1 was correlated with Na^+^/K^+^ATPase activity, an antiporter enzyme with important roles in maintaining resting potential, availing transport, regulating cellular volume, and as a signal transducer/integrator to regulate MAPK pathway, ROS, as well as intracellular calcium. As ions, nutrients, and liquid enter the lens from the aqueous humor, Na^+^/K^+^-ATPases in the lens epithelial cells pump ions out of the lens to maintain appropriate lens osmolarity and volume, with equatorially positioned lens epithelium cells contributing most to this current. The activity of the Na^+^/K^+^-ATPases keeps water and current flowing through the lens from the poles and exiting through the equatorial regions. Similar to mice, 58 kDa and 120 kDa isoforms of Panx1 were detected, further Cx53 and Cx50, but not Panx2, Panx3 or Cx40. Hypoosmotic, but not a hyperosmotic solution triggered pannexin- and connexins-mediated ATP release increasing Na^+^/K^+^ATPase activity. This activity was pharmacologically reduced by the connexin inhibitor 18a-glycyrrhetinic acid (AGA), and abolished when AGA was combined with the Panx1 specific inhibitor probenecid, or when apyrase was added to catalyze the hydrolysis of ATP. Using the exocytosis inhibitor N-ethylmaleimide (NEM), the authors ruled that ATP is released via exocytosis. Using the purinergic P2 receptor antagonist reactive blue-2 and pertussis toxin (PTX), the authors showed that the increased Na^+^/K^+^-ATPase activity under hyposmotic stimulation is most probably mediated by G-protein coupled P2Y receptor activation. Thus, increased Na^+^/K^+^ATPase activity of the epithelium during a hyposmotic challenge was prevented when either ATP release or extracellular ATP accumulation was suppressed, or P2Y receptors were inhibited. This result correlated Panx and connexin activities with ATP release, most likely due to the channels’ mechanosensitivity (Bao et al., 2004a,b), in response to swelling of the lens cells during the hyposmotic stimulation.

Pannexin/connexin-mediated ATP release during hyposmotic stress was linked to TRPV4 channel activation by Shahidullah et al. (2012). The TRPV4 encoded protein is a Ca^2+^-permeable, nonselective cation channel that is involved in the regulation of systemic osmotic pressure. Like other members of the TRP superfamily, TRPV channels can be activated through seemingly disparate mechanisms. In the lens epithelium, TRPV4 inhibitors prevented ATP release and propidium iodide uptake of the lens epithelium and an agonist elicited ATP release and propidium iodide uptake even under isosmotic conditions. Similarly, in esophageal epithelial cells it was shown that TRPV4 activation triggers Cx43- and Panx1-mediated ATP release (Ueda et al., 2011), and in the airway epithelium TRPV4 transduced the membrane stretch signal upon leading to hypotonicity-induced, Panx1-mediated ATP release (Seminario-Vidal et al., 2009). Since the TRPV4 channel is permeable for Ca^2+^ ions, the activation of TRPV4 caused an increase of intracellular Ca^2+^ in response to the hyposmotic challenge, which was eliminated by the two TRPV4 antagonists and elicited by the agonist under isosmotic conditions. The intracellular Ca2^+^ rise was also prevented when the bathing solution was calcium free. Thus, the Ca^2+^, which is entering the cells through activated TRPV4 channels during the hyposmotic challenge, might in turn activate Panx1 channels (Locovei et al., 2006b) or connexin hemichannels like Cx32 (De Vuyst et al., 2006) or Cx43 (Retamal et al., 2007). Since inhibition of TRPV4 prevented ATP release during hyposmotic stress, no increased Na^+^/K^+^ATPase activity was evoked. Summing up, the results reported suggest that Panx1 together with connexins, ATP and purinergic receptors participates in osmotic regulation, through modulation of Na^+^/K^+^ATPase activity.

Panx1 was also identified in blood endothelial cells of the *tunica vasculosa lentis* (Dvoriantchikova et al., 2006b), an extensive capillary network deriving from the *vasa hyaloidea*, spreading over the posterior and lateral surfaces of the lens, which disappears after birth (Skapinker and Rothberg, 1987; Barishak, 1992). There, Panx1 was mostly found at the luminal side of the blood capillaries. No co-localization with Cx50 was detected. Panx1 channels, alongside with connexins, are localized in a strategic position alongside connexins with potential to participate in differentiation and lens homeostasis. With Panx1 channels known to play a significant role in cell death, it is tempting to speculate about a role in the retinopathy of prematurity (Pau, 2008). It remains to be demonstrated whether pharmacological blocking of Panx1 in patients with this condition can modulate the disproportional regression of the *vasa hyaloidea* by cell death and the proliferation of retinal vessels, causing fibrovascular tissue to overgrow the remnants of retina-attaching hyaloidal structures through the vitreous cavity up to the *tunica vasculosa lentis*.

## Panx1 and aqueous humor outflow: implications in glaucoma

A potential role of Panx1 channels in pressure regulation of the aqueous humor outflow pathway has emerged recently (Li et al., 2010, 2012). Two parallel pathways mediate the outflow of the aqueous humor. In the uveoscleral pathway, aqueous humor is flowing through ciliary muscle bundles and subsequent passage to pressure-insensitive routes (Toris et al., 2008). The pressure-sensitive trabecular meshwork pathway comprises the trabecular meshwork, juxtacanalicular tissue, the inner wall of the Schlemm’s canal, collector channels and aqueous veins (Gong et al., 1996; Goel et al., 2010). Cells of the outflow pathway are important for the regulation of the outflow, with trabecular meshwork cells playing a critical role (Francis and Alvarado, 1997). Among the substances released by trabecular meshwork cells are components of the extracellular matrix, like metalloproteinases (MMPs) and their inhibitors modeling the extracellular matrix and therewith the outflow resistance (Aga et al., 2008). The release of MMP-2 is regulated by A1 adenosine receptors on trabecular meshwork cells (Shearer and Crosson, 2002), leading to reduced outflow resistance and reduced intraocular pressure (IOP). Inflow and outflow pressure regulation involves adenosine receptor pathways. The A3 receptor activation enhances the intraocular pressure by activating chloride channels on nonpigmented ciliary epithelial cell that secrete chloride and enhance aqueous humor formation (Mitchell et al., 1999; Avila et al., 2001). Both act in concert and are potential targets to reduce IOP in glaucoma patients. It is tempting to speculate whether a feedback control mechanism exists involving Panx1 released ATP that is metabolized by ecto-ATPases to adenosine (Shearer and Crosson, 2002; Husain et al., 2007). Results that provided first evidence were quantitative RT-PCR experiments demonstrating that Cx26, Cx31 and Cx43 were expressed in trabecular meshwork cells, with Panx1 and Cx43 being equally expressed in the human trabecular meshwork cell line TM5. In human explant-derived primary trabecular meshwork cells Cx43 levels were 10-fold higher compared to Panx1, whereas the expression of the P2X7-R is about a 100-fold lower. Applying 21 different inhibitors, Li et al. (2010) showed that Panx1 channels, connexin hemichannels, and P2X7-R are important in swelling-activated ATP release from trabecular meshwork cells, with Panx1 and connexins accounting for about 40% each of the released ATP, and P2X7-R for about 16% or 30% when Panx1 and connexin hemichannels were not blocked simultaneously. This supports a role of Panx1, most likely in cooperation with connexins and P2X7-R in modulating the pressure of the outflow pathway. Further, in the bovine ciliary epithelium, quantitative RT-PCR experiments revealed the expression of Panx1, Cx40, Cx43 and P2X7-R in mixed primary cultures of nonpigmented and pigmented ciliary epithelial cells and transformed nonpigmented and pigmented epithelial cells. Using 11 different inhibitors, Li et al. (2010) showed that in these cells during hypotonicity-triggered ATP release Panx1 and connexins contribute to the ATP release, but that P2X7-R-mediated ATP release was insignificant, whereas 20% of the ATP release was vesicular.

A role of Panx1 in modulating pressure is potentially of significant clinical relevance. Glaucoma represents a group of ocular disorders with multi-factorial etiology united by a clinically characteristic IOP associated optic neuropathy and is a leading cause for blindness. The treatment that delays the onset and slows the progression of glaucomatous blindness is reducing IOP. Evidence is accruing that Panx1 might be directly involved in the pathology of glaucoma. It has been shown that ATP levels are elevated with increased IOP (Resta et al., 2007; Li et al., 2011), even in correlation with the magnitude of increased pressure in the vitreous of patients with acute angle closure glaucoma (Zhang et al., 2007). It has been hypothesized that excess extracellular ATP might lead to neuronal death, since stimulation of P2X7-R on RGCs leads to an increase of the cytosolic Ca^2+^ concentration with subsequent excitotoxic cell death (Zhang et al., 2005). The P2X7-R is of special importance, as the receptor can also initiate inflammatory responses, which play a role in glaucoma (reviewed in Krizaj et al., 2014). Also, RGCs were rescued during rapidly increased ocular pressure by apyrase-mediated dephosphorylation of ATP and by inhibiting purinergic receptors (Resta et al., 2007). In accordance, mechanical perturbations, e.g., due to increased hydrostatic pressure, cell swelling or stretching, shear stress, are most efficiently triggering ATP release (discussed in Reigada et al., 2008). One study linked a possible involvement of Panx1 in glaucoma pathogenesis (Reigada et al., 2008) to Panx1 as a mechanosensitive ATP-release channel (Bao et al., 2004a,b). Using *ex vivo* bovine eyecup preparations, the authors showed that 10 min of increased pressure of 20 mmHg above the atmospheric pressure led to physiological ATP release in the vitreal chamber that declined back to the baseline within 30 min. In contrast, applying 70 mmHg led to a constant increase of the ATP levels throughout a 60 min experiment. Furthermore, the authors demonstrated that the amount of ATP strongly correlated with the applied pressure. Carbenoxolone, 5-nitro-2-(3-phenylpropylamino) benzoic acid (NPPB; Silverman et al., 2008), but not mefloquine, reduced the ATP release by about 90%, leading to the author’s conclusion of a Panx1-mediated ATP release. Since mefloquine efficiently inhibits Panx1 channel in the nM concentration range (Iglesias et al., 2009b), but failed to reduce the pressure-induced ATP-increase in this study, the results raise the question whether connexin hemichannels or other ATP release mechanisms, like vesicular release were involved. Further, NPPB can inhibit other channels that have been associated with ATP release (discussed in Reigada et al., 2008), like volume sensitive channels (Furukawa et al., 1998), voltage-dependent anion channels (VDAC; Sabirov et al., 2001) and the cystic fibrosis transmembrane conductance regulator (CFTR; Cuthbert, 2001). Using isolated rat RGCs and applying blocker pharmacology, a subsequent study proved that RGCs themselves mediate mechanosensitive ATP release, as they respond upon bathing in hypotonic solutions to swelling-induced mechanical stress with rapid and sustained Panx1-mediated ATP release, which in turn autostimulates P2X7-R on RGCs (Xia et al., 2012). In mixed retinal cultures, the hypotonicity-induced ATP levels rapidly rose to a transient peak and afterwards declined to a steady state, indicating a higher rate of ATP breakdown than ATP release. In contrast, in isolated RGCs the ATP levels constantly rose during the recording time, indicating less ecto-ATPase activity compared to the mixed cultures. Also, stretching mixed retinal cells or isolated RGCs lead to ATP release in a Panx1-dependent manner. Subsequent whole-cell patch clamp recordings from RGCs in mixed retinal cultures and isolated RGCs revealed that swelling induced ATP release activates P2X7-R on RGCs. In turn, the ecto-ATPase apyrase, probenecide or carbenoxolone or the P2X7-R antagonists A438079, AZ 10606120 and zinc reduced the swelling-activated currents. Since Panx1 and P2X7-R are expressed on RGC neurites, Xia et al. (2012) reasoned that the ATP release and P2X7-R autostimulation take place in the RGC axons. These results are unexpected, since other studies have shown that increased IOP first affects the dendritic field size, number of synapses, light-evoked responses and leads to abnormalities in dendritic arbors prior to a reduction of axon thickness and deformation of the optic nerve head (reviewed in Krizaj et al., 2014). Interestingly, Panx1-mediated ATP release seems to be important for RGCs cells for their regulatory volume decrease. Under control conditions during bathing in hypotonic solution, the RGCs initially swell to a peak size, but after 30 min decrease their volume again due to regulatory volume decrease, which was reduced by about 60% when probenecide was applied, indicating a beneficial contribution of Panx1 channels in this context. Still, in an event of chronic increased IOP, Panx1 may mediate the release of excessive ATP amounts leading to harmful autocrine stimulation of P2X7-R that drives the whole system in a pathological state, leading to the P2X7-R/Panx1 “death-complex”. Thus, in accordance with being localized on neurites, this putative damaging signaling pathway can cause RGC death by damaging axonal processes, a possible mechanism in glaucoma pathology. A novel finding is that astrocytes isolated from optic nerve heads upregulated Panx1 expression and increased ATP release with sustained stretch (Beckel et al., 2014). This suggested a glia cell based mechanism for maintaining elevated extracellular ATP upon sustained pressure induced mechanical strain. Further, Beckel et al. concluded that physiological consequences could be either beneficial or detrimental for RGCs, depending on the relative levels of adenosine or P2X7-R/Panx1. Other mechanosensitive (like TRPV4) or inflammatory factors in glaucoma exist and have been recently reviewed (Krizaj et al., 2014).

## Panx1 in the cornea: implications for wound healing

IHC experiments revealed that Panx1 is also expressed in the cornea of mice (Mayo et al., 2008). In this study, the primary purpose was to analyze the role of P2X7-R in the repair of *in vivo* corneal epithelial debridement wounds and in the structural organization on the corneal stroma. After epithelial debridement was performed on P2X7-R^−/−^ and wild-type mice, light microscopic, immunohistochemical, and electron microscopic analysis showed that Panx1 was localized between cells with a certain distance distal to the leading edge of epithelial debridement of mice corneas. In contrast, Panx1 was detected throughout the epithelium and at the leading edge of the injury in WT animals. This indicated that the absence of P2X7-R alters the localization of Panx1 in the cornea. Further, in P2X7^−/−^ mice the rate of wound repair was negatively affected. The absence of Panx1 at the wound edge is in line with another study about Panx1 in skin development and wound healing (Penuela et al., 2014a).

## Challenges in pannexin research

Research addressing the (patho)physiological roles of Panx1 needs to overcome biological, technical and logistical challenges. A significant biological challenge derives from the complex interactions of Panx1 with major signaling pathways involving ionotropic and metabotropic receptors (Isakson and Thompson, 2014), the inflammasome (Adamson and Leitinger, 2014) cell death pathway (Jackson et al., 2014), or the cytoskeleton (Boyce et al., 2014). The capacity of Panx1 to undergo multiple interactions is exciting, suggesting clinically relevant roles in many physiological and pathophysiological settings. Dissecting complex networks of interactions has high priority and will guide our understanding of the (patho)physiology of Panx1 in the eye and beyond.

Pharmacological manipulation of Panx1 is a second challenge despite the significant advances made in recent years. Blocking peptides like ^10^Panx1 (Pelegrin and Surprenant, 2006), peptides interfering with Panx1 in a specific context like anoxia (Weilinger et al., 2012), or pharmacological blockers like probenecid (Silverman et al., 2008), mefloquine (Iglesias et al., 2009b), or more recently the food dye FD&C Blue No. 1 (Wang et al., 2013) have greatly improved our understanding of Panx1. These studies were mostly performed in the context of single cell types or oocytes and all compounds used acted by closing the Panx1 channel. However, it might be beneficial in certain conditions to open Panx1 channels when ATP efflux is needed. Further, there is a demand for selective agonists/antagonists to discriminate between the three Panx channels and connexin hemichannels frequently co-expressed in complex tissues like the eye.

The availability of Panx1 KO mouse models has greatly contributed to the understanding of Panx1 functions *in vivo*. A challenge emerging from mouse models and KO cell lines is the question of compensatory upregulation of other Panxs or connexin hemichannels. Recently, Panx3 has been shown to be upregulated in the arterial walls and skin of Panx1 knock out mice (Lohman and Isakson, 2014; Penuela et al., 2014a). In case compensatory regulation is more common, proving functional compensation will critically depend on specific blockers not available at present. Rational designed drugs to selectively target Panx proteins could derive from mimetic peptides with less cross-inhibition or pharmacological screens of natural compounds (Grek et al., 2014; Saez and Leybaert, 2014).

Dissecting complex interactions and solving pharmacological shortcomings are of general concern. A specific concern for vision and eye research is choosing appropriate animal models. While vertebrate eyes share a general anatomy and physiology, visual capabilities are distinct, frequently reflecting functional adaptations to habitats and/or behaviors. Complementary models are needed to weight advantages over disadvantages of each species when investigating the roles of Panxs in the eye. Already distinct functional roles of Panx1 in the mouse and zebrafish retina have emerged, reflecting unique properties of the two species (Kranz et al., 2013; Vroman et al., 2014). Beyond doubt, studies in mouse and fish models have and will aid our understanding of fundamental roles of Panx1 in the visual system, but to build a more generalized view of Panx functions complementary studies in other higher vertebrates like rats and rabbits, or in color vision competent non-human primates will be needed. Recent advances in genome engineering technologies based on transcription activator-like effector nucleases (TALEN) or the RNA-guided Cas9 nuclease (Cas9/CRISPR) have been successfully used to generate first functional knock downs or knock outs in a wide range of species including non-human primates (Mali et al., 2013a,b; Liu et al., 2014a,b; Niu et al., 2014; Yang et al., 2014) making targeting Panx1 in higher vertebrates an experimental option.

## Concluding remarks

Panx1 research in the eye has moved from early expression and localization studies to functional studies. This research is currently driven by the availability of knock out animal models, sophisticated *ex vivo* and *in vitro* preparations suitable to address the emerging roles of Panx1 in a physiological or pathological context, and the potential implication of Panx1 in leading causes of blindness. There is no confirmed unifying theme to be highlighted yet. Instead, we wish to communicate that Panx1 channels are highly versatile channels. Functions are mediated by the distinct localization of Panx1 proteins in different cell types, multiple protein interactions, as well as post-translational modifications. Since Panx1 channels operate at the crossroad of major signaling pathways, it is tempting to speculate that long lasting changes to this channel have the potential to impact physiological functions by altering major signaling pathways. ATP release, as found in the humor outflow system or as a mediator of cross talk between neurons and microglia, has the potential to be the unifying molecular function, connecting biophysical properties of Panx1 channels to ATP mediated signaling. However, this generalization might not be fully true. Bipolar cells like CB3a cells do not release ATP in any known physiological or pathological context. Further, HC release ATP, but it is the pH shift evoked by the breakdown of ATP to adenosine that alters neuronal communication. Since insight into the relevant molecular, cellular and physiological roles of Panx1 in conditions as diverse as primary processing of visual information in the outer retina or IOP regulation is emerging, we expect soon advances in conceptual insight related to the different roles of Panx1 in this physiologically and clinically important sensory organ.

## Conflict of interest statement

The authors declare that the research was conducted in the absence of any commercial or financial relationships that could be construed as a potential conflict of interest.
